# Life satisfaction in children: an analysis of the psychometric properties of the SWLS-C scale by Diener

**DOI:** 10.1186/s40359-025-02690-4

**Published:** 2025-04-15

**Authors:** Marly Johana Bahamón, Lorena Cudris-Torres, José Julián Javela, Andrea Ortega-Bechara, Andrés Cabezas-Corcione, Álvaro Barrios-Nuñez, Valmore Bermúdez

**Affiliations:** 1 Universidad del Sinú, Montería, Colombia; 2 Universidad de La Costa, Barranquilla, Colombia; 3 Clínica General del Norte, Barranquilla, Colombia; 4 Centro de Investigaciones en Ciencias de La Vida, Universidad Simón Bolívar, Barranquilla, Colombia

**Keywords:** Life satisfaction, Subjective well-being, Adolescents, SWLS-C, Psychometric properties

## Abstract

**Background:**

The psychometric properties of the Satisfaction with Life Scale for Children (SWLS-C) are presented (Gadermann et al., Soc Indic Res 96:229–47, 2010).

**Methods:**

A total of 1,242 children participated (48.3% boys and 51.7% girls), aged between 10 and 14 years (*M* = 12.19, *SD* = 1.54). A confirmatory factor analysis (CFA) was conducted to evaluate the factorial structure of the SWLS-C, assuming a unidimensional model using the diagonally weighted least squares (DWLS) estimation method due to the lack of normality assumption. Internal consistency was calculated using Cronbach's alpha and McDonald's omega, and convergent validity was assessed using the Spearman Rho coefficient between the total SWLS-C score and the Life Satisfaction evaluations from the SLSS and the Perceived Social Support scale by Zimet. The effect of age and sex variables was estimated as predictors through a multivariate regression model.

**Results:**

The fit and goodness indices (RMSEA =.058, CFI =.098, TLI =.096, GFI =.099).

**Conclusions:**

Show that the scale is valid and reliable for assessing life satisfaction in Colombian children and adolescents.

**Supplementary Information:**

The online version contains supplementary material available at 10.1186/s40359-025-02690-4.

## Background

The study of subjective well-being has progressively gained interest as a key area in understanding health from a broader perspective that extends beyond the traditional focus on disease and the mere presence or absence of symptoms. Therefore, its analysis constitutes a fundamental component of the concept of health, as individuals should have the ability to experience well-being throughout their lives [1, 2].

One of the core components of subjective well-being is life satisfaction, which is defined as the global judgment an individual makes about the quality of their life based on their own unique criteria [3] While this construct has been extensively studied in adults, research on life satisfaction in children and adolescents has not progressed at the same pace, despite its potential impact on long-term development and mental health [4, 5]. This gap presents significant challenges related to cognitive development, emotional stability, and identity formation in younger populations. Unlike adults, children and adolescents are still undergoing developmental changes, which may influence how they perceive their well-being and, consequently, how they respond to standardized questionnaires [6, 7]. For these reasons, it is crucial to have assessment instruments specifically designed for children and adolescents, ensuring that the items are both comprehensible and developmentally appropriate [8],

In this regard, the Satisfaction with Life Scale (SWLS) [6] has become one of the most widely used instruments for assessing life satisfaction in adults, with validation studies conducted across various countries worldwide [7–9]. However, to adapt this scale for younger populations, researchers developed a modified version known as the Satisfaction with Life Scale for Children (SWLS-C) [10, 11]. This adaptation is essentia l for accurately measuring life satisfaction in children and adolescents, enabling consistent comparisons across developmental stages.

### Cross-cultural adaptations and psychometric validity of the SWLS-C

Satisfaction with Life Scale for Children (SWLS-C) has emerged as a valuable instrument for health professionals investigating mental health from a positive psychology perspective. For this reason, it has undergone various cross-cultural adaptation and validation processes, yielding satisfactory results across multiple cultural and contextual settings, which is essential for ensuring its reliability and validity. Within this framework, numerous studies have examined the psychometric properties of the SWLS-C in diverse populations of children and adolescents worldwide.

In Chile, a cross-cultural adaptation and validation study of the SWLS-C in Chilean adolescents [1], conducted through a rigorous translation and back-translation process, demonstrated that the Chilean version of the scale exhibits strong psychometric properties. First, reliability was assessed, measuring the degree of correlation among the scale's items. In this context, values above 0.70 are considered acceptable, while values above 0.80 are deemed optimal for psychological measurement instruments. The internal consistency of the SWLS-C in this study was α = 0.815, indicating adequate reliability. Moreover, convergent validity analysis revealed a significant correlation with the Children’s Depression Inventory (CDI), supporting the SWLS-C’s ability to validly assess life satisfaction in adolescents.

Regarding confirmatory factor analysis (CFA), the fit indices obtained confirmed an adequate model fit. The RMSEA (Root Mean Square Error of Approximation), which evaluates the discrepancy between the hypothesized model and the observed data, yielded a value of 0.05, indicating a good fit, as values below 0.08 are considered acceptable. Additionally, the CFI (Comparative Fit Index), which assesses the model’s quality relative to a baseline model with no relationships among items, produced a value of 0.95, suggesting a good fit, since values above 0.90 indicate an adequate factorial structure.

Another notable study assessed the factorial invariance of the SWLS in adolescents from Spain and Portugal using multi-group confirmatory factor analysis (MG-CFA) [12]. The results demonstrated high internal consistency in both versions (Spanish and Portuguese) and supported the unidimensional structure of the scale. The fit indices obtained included a CFI of 1.00, a TLI (Tucker-Lewis Index) of 1.00, and an RMSEA of 0.004 for the Spanish sample, while the Portuguese sample reported a CFI of 0.998, TLI of 0.996, and RMSEA of 0.035. However, scalar invariance was not fully achieved, indicating differences in item interpretation between adolescents from the two countries [12].

In the Asian context, studies conducted in South Korea [13].have replicated consistent findings regarding the unidimensional structure of the SWLS-C and its high reliability, as evidenced by a Cronbach’s alpha coefficient of 0.93, indicating excellent internal consistency. The Korean version of the scale was administered to 653 primary school students, and confirmatory factor analyses demonstrated an adequate model fit, further supporting the psychometric robustness of the scale in this population. The results included a CFI of 0.98, TLI of 0.97, and RMSEA of 0.04, reinforcing the stability of the factorial structure in the Korean sample.

The CFI (Comparative Fit Index), which compares the fit of the theoretical model to a baseline model with no relationships among items, reached a value of 0.98, suggesting that the proposed unidimensional structure adequately represents the observed data. Similarly, the TLI (Tucker-Lewis Index), which evaluates the quality of the model while penalizing unnecessary complexity, obtained a value of 0.97, indicating that the model is parsimonious and well-fitted. Meanwhile, the RMSEA (Root Mean Square Error of Approximation), which measures the discrepancy between the theoretical model and the observed data, yielded a value of 0.04, indicating excellent fit, as values below 0.05 reflect minimal discrepancy.

In Canada, Gadermann et al. [10] adapted the SWLS-C and evaluated its validity in primary school students, demonstrating high internal consistency (α = 0.84), measurement invariance across gender and time, and significant associations with optimism, self-efficacy, and depression. Additionally,.Emerson et al., (2018) confirmed cross-cultural measurement invariance between English-speaking children and other cultural/linguistic groups, highlighting the applicability of the SWLS-C in multicultural settings [14].

Similar studies conducted in British Columbia examined the measurement invariance of the SWLS-C with respect to gender and time in primary school students [15]. Likewise, in the context of ethnocultural diversity, measurement equivalence was demonstrated between English-speaking children and other linguistic/cultural groups [14], confirming that the SWLS-C consistently measures life satisfaction across diverse cultural backgrounds.

A cross-cultural adaptation and validation study conducted in France examined the factorial structure and measurement invariance of the French adaptation of the SWLS in children and adolescents, focusing on differences related to age and gender. The results supported the unidimensional structure of the scale and confirmed that the French adaptation of the SWLS is suitable for use with children and adolescents in both cross-sectional and longitudinal studies, enabling comparisons across genders, time periods, and age groups [16].

### Psychometric properties and advanced analyses

Beyond its factorial structure, various studies have further examined the psychometric properties of the SWLS-C in specific populations. In Norway [17], the psychometric properties of the SWLS-C were evaluated in a sample of 1,073 Norwegian adolescents aged 13 to 18 years, with a balanced gender distribution (51.2% female). Confirmatory factor analyses (CFA) supported a unidimensional structure, although it was necessary to account for residual correlations between items 4 and 5, reflecting a specific relationship in assessing retrospective aspects of life satisfaction. The internal consistency was high (α = 0.87), and the composite reliability (CR) was 0.87 for the overall sample, with 0.86 for males and females separately. Convergent validity was adequate, with an Average Variance Extracted (AVE) of 0.57, and significant positive correlations with self-esteem (r = 0.66) and negative correlations with anxiety (r = − 0.58) and depressive symptoms (r = − 0.54).

Regarding measurement invariance, the configural and metric invariance of the SWLS-C was confirmed across genders, indicating that the scale measures life satisfaction equivalently in boys and girls. However, scalar and residual invariance were not fully achieved, suggesting differences in item intercepts and residual variances between genders. Overall, these findings support the validity and reliability of the SWLS-C as an appropriate instrument for assessing life satisfaction in Norwegian adolescents, while also highlighting the need for further research into modified factorial structures and measurement invariance in other adolescent populations.

In Turkey, a psychometric evaluation of the SWLS-C was conducted among university students [18]. The findings confirmed the high reliability and validity of the scale across different age groups and contexts, with confirmatory factor analysis (CFA) fit indices demonstrating an adequate model fit (CFI = 0.97, RMSEA = 0.05), reinforcing the applicability of the SWLS across diverse populations.

Another notable study was conducted in Indonesia by Natanael et al., (2024), who assessed the psychometric properties of the Indonesian version of the SWLS in a sample of 1,154 university students aged 17 to 27 years (*M* = 19.86, *SD* = 1.22), of whom 72.8% were female [19]. The analysis confirmed that the scale met the assumptions of unidimensionality (variance explained by the model = 80.2%) and local independence (residual correlations between items < 0.30), supporting its construct validity.

Additionally, CFA results demonstrated a strong model fit (χ^2^(5) = 44.749, CFI = 0.992, TLI = 0.983, RMSEA = 0.067, SRMR = 0.014), further validating the unidimensional model. In terms of reliability, the SWLS-C exhibited high internal consistency, with Cronbach’s alpha of 0.94, person reliability of 0.97, and item reliability of 1.00, indicating excellent reliability. The person separation index was 3.86, and the item separation index was 9.50, both significantly above the recommended threshold (> 3), demonstrating strong discriminatory power.

Furthermore, an analysis of differential item functioning (DIF) revealed low-level differences in items 3 and 5, where male participants exhibited a higher tendency to agree with item 5 (desire to change one’s life), while female participants showed greater agreement with item 3 (self-evaluation of life satisfaction). Finally, a response scale analysis recommended adjusting the original 7-point format to a 5-point format, as this modification enhanced psychometric performance and improved clarity for participants. Overall, these results support the validity, reliability, and measurement fairness of the Indonesian version of the SWLS, establishing it as a suitable tool for future research on life satisfaction among Indonesian university students.

### The Importance of cultural and linguistic adaptation

The process of cultural and linguistic adaptation is crucial for ensuring the semantic equivalence and cross-cultural validity of the SWLS-C. Studies, such as that of Busubul et al.2023 in Indonesia, have highlighted the importance of this process by identifying subtle differences in item interpretation depending on the cultural context [20]. Additionally, the convergent validity of the SWLS-C with other well-being measures, such as the Positive and Negative Affect Schedule (PANAS), further supports its usefulness in assessing subjective well-being in child populations across diverse settings.

Despite the robust evidence supporting the SWLS-C in various contexts, as with other cross-cultural adaptation processes, it is essential to consider potential cultural biases that may influence item interpretation. In collectivist cultures, such as Indonesia, life satisfaction may be more closely linked to family or community harmony than to individual judgments, potentially affecting the construct validity of the scale. Additionally, previous adaptations in non-Western cultures have faced challenges in replicating the original unidimensional factorial structure [12], emphasizing the need for rigorous evaluations of semantic and conceptual equivalence for each item. These cultural and methodological differences pose a challenge for the generalization of psychometric findings across different countries, underscoring the need for localized validation studies that assess the SWLS-C based on the sociocultural characteristics of each population, such as Colombian children.

In this regard, despite the abundant international evidence, Colombia lacks sufficient studies that thoroughly analyze the psychometric properties of the SWLS-C in child populations. Given the importance of evaluating subjective well-being from early ages and the need for culturally adapted instruments, this study aims to assess the factorial structure, reliability, convergent validity, and the influence of sociodemographic variables on SWLS-C scores in a sample of Colombian children.

This study specifically aims to evaluate the psychometric properties of the SWLS-C in Colombian children. More precisely, it seeks to analyze its factorial structure, assess its reliability and convergent validity, and explore the influence of sociodemographic variables, such as gender and age, on life satisfaction scores. Through this research, we aim to provide empirical evidence on the suitability of the SWLS-C in child populations and its utility in measuring subjective well-being in Latin American populations.

## Methods

### Participants and procedure

A total of *N* = 1,242 children participated in the study, including 600 boys (48.3%) and 642 girls (51.7%), aged between 10 and 14 years (*M* = 12.19, *SD* = 1.54). Regarding educational level, the minimum schooling was 3 years, while the maximum was 10 years (*M* = 6.61, *SD* = 1.54). In terms of family structure, most children belonged to nuclear families (53.0%), followed by extended families (26.6%) and single-parent families (12.9%). The remaining percentage corresponded to children from blended families or cases with no reported family structure.

Participants were recruited by senior psychology students, who had been previously trained in the administration of assessment instruments. The study was conducted in educational institutions located in urban areas of northern Colombia, specifically in low- and middle-income sectors. School selection was based on accessibility, institutional approval, and willingness to participate in the research. The sample was not designed to be nationally representative of the Colombian child population, which is acknowledged as a limitation of the study. Inclusion Criteria: Children aged 10 to 14 years enrolled in the selected schools and signed informed consent from parents or legal guardians and child assent to participate. Exclusion Criteria: Diagnosed cognitive impairments or neurodevelopmental disorders that could affect comprehension of the assessment instruments and lack of parental consent or child assent to participate. Research assistants personally visited the parents to obtain consent and provide all relevant information about the study. Subsequently, children were individually contacted to request their voluntary participation and to administer the assessment instruments in a controlled environment within their educational institutions.

### Instruments

*Satisfaction with Life Scale for Children (SWLS-C)* [6, 11]: Life satisfaction is a key component of subjective well-being, reflecting an individual's overall cognitive evaluation of their life quality based on personal standards and expectations. This scale assesses life satisfaction through the global evaluation individuals make of their lives. It has a response scale ranging from 1 to 5, where 1 is"strongly disagree"and 5 is"strongly agree."The items are presented as follows: (a) In most aspects, my life is close to how I would like it to be, (b) Things in my life are excellent, (c) I am happy with my life, (d) So far, I have gotten the important things I want in life, (e) If I could live my life again, I would have it the same way. The absolute residual values in a unidimensional model reported by the authors vary between 0.0007 and 0.034, and the factor loadings of the five items determined by factor analysis are high (equal to or greater than 0.70). The Cronbach's alpha coefficient is 0.86, and the ordinal alpha coefficient was 0.90.

*Perceived Social Support Scale (MSPSS)* [21] Perceived Social Support Scale (MSPSS), [22] validated in Colombia. Social support is a fundamental psychological resource that enhances well-being by providing emotional, instrumental, and informational assistance. This is a 12-item instrument that measures perceived adequacy of social support from three sources: family members (items 3, 4, 8, and 11), friends (items 6, 7, 9, and 12), and significant others (items 1, 2, 5, and 10). These 12 items are rated on a 7-point Likert scale from strongly disagree (1) to strongly agree (7). The total score corresponds to the sum of the scores for each item answered, with higher scores indicating greater perceived social support. The validity indices found have been satisfactory (RMSEA = 0.049, NNFI = 0.946, CFI = 0.975, AIC = 31.680). The internal consistency coefficient has shown satisfactory reliability indices (Global social support α = 0.84; family support α = 0.82; friends support α = 0.84; others support α = 0.75).

*The Student's Life Satisfaction Scale (SLSS)* [23, 24], translated and validated in Latin America. Life satisfaction in children and adolescents is a crucial indicator of overall well-being, encompassing their evaluations of various life domains, including family, school, and peer relationships. This scale contains seven items that assess global aspects of schoolchildren's lives, presented as follows: 1) My life is going well, 2) My life is the way I want it to be, 3) I would like to change many things in my life, 4) I would like to have a different kind of life, 5) I have a good life, 6) I have what I want in life, and 7) My life is better than most other kids my age. The response scale used is a 5-level Likert scale (0 = Strongly disagree; 4 = Strongly agree). Items 3 (I would like to change many things in my life) and 4 (I would like to have a different kind of life) are reverse-scored to obtain a total score. The scores were averaged to obtain the overall score representing overall life satisfaction. The reliability coefficient reported by the authors is 0.86, and the validity indices indicate good fit (RMSEA = 0.12, TLI = 0.97, CFI = 0.98).

### Ethical considerations

This study was approved by the Ethics Committee of Universidad del Sinú (Act No. 003, approval code UNISINU- 003–2024, issued on April 30, 2024), ensuring compliance with international ethical standards, including the principles of autonomy, beneficence, and justice established in the Declaration of Helsinki. Participation in the study was voluntary, and ethical safeguards were implemented to protect the rights and well-being of all participants. Informed consent was obtained from parents or legal guardians, and assent was secured from the children, ensuring that they understood the study’s objectives, the voluntary nature of their participation, and their right to withdraw at any time without consequences.

Confidentiality and anonymity were strictly maintained, with all identifying information removed and securely stored for scientific purposes only. Assessments were conducted in a controlled environment within the participants’ educational institutions, and researchers trained in psychological assessment in child populations ensured that the administration of the instruments was ethical and developmentally appropriate. Additionally, a safety and referral protocol was established to ensure that any participant experiencing significant emotional distress during the assessment was referred to mental health services available at the collaborating institutions. These measures ensured that the research was conducted with the highest respect for the rights, dignity, and safety of the children involved.

### Statistical analysis

A confirmatory factor analysis (CFA) was conducted to evaluate the factorial structure of the SWLS, assuming a unidimensional model where all items correspond to a single factor. The univariate normality test was estimated using the Kolmogorov–Smirnov-Lilliefors test, and the multivariate normality test using Mardia's test [25]. Given that the results of these analyses indicated that the assumption of normality was not met (Kolmogorov–Smirnov test, *p* < 0.001; Mardia’s Skewness = − 1.550 to − 0.662, *p* < 0.0001; Mardia’s Kurtosis = 2.69—0.148*, p* < 0.0001), the diagonally weighted least squares (DWLS) estimation method was chosen.

DWLS was selected instead of maximum likelihood (ML) because ML assumes multivariate normality, and when this assumption is violated, it can lead to biased parameter estimates and incorrect standard errors. DWLS is a robust estimation method recommended for ordinal data and non-normally distributed continuous variables, as it provides more reliable parameter estimations and robust standard errors under these conditions. Given that the SWLS consists of Likert-type response items, DWLS was the most appropriate estimation method for this study. To estimate the model parameters, all factor loadings were freely estimated, allowing the data to determine the strength of the relationship between each item and the latent factor. This approach ensures that the model optimally reflects the variance explained by each item without imposing arbitrary constraints on the factor loadings.

To assess the psychometric properties of the SWLS, internal consistency was evaluated using Cronbach’s alpha and McDonald’s omega. Cronbach’s alpha was included for comparability with previous studies, despite its assumption of tau-equivalence, which may not always hold in psychological measures. McDonald's omega was also computed as it provides a more robust estimation of reliability by accounting for differences in factor loadings across items. Convergent validity was examined through a Spearman’s rho correlation analysis between the total SWLS score and life satisfaction assessments from the SLSS, as well as the Perceived Social Support Scale developed by Zimet.

The influence of age and sex on life satisfaction was analyzed using a multivariate regression model rather than a group comparison approach. This methodological choice was based on the advantages of regression analysis, which allows for the assessment of age as a continuous variable, avoiding arbitrary categorization. Additionally, this approach enables the simultaneous control of multiple predictors and the examination of potential non-linear (curvilinear) relationships, which is particularly relevant in developmental studies.

In the regression model, age was included as both a linear and quadratic predictor to account for potential curvilinear effects. To mitigate multicollinearity, the age variable was centered by subtracting the sample mean. Sex was coded as male = 1 and female = 0. The model assumes that residuals follow a normal distribution with a mean of 0 and variance σ_ε^2 (ε_i∼N(0σ_ε^2)).

Forward selection was chosen over backward selection and stepwise regression due to its advantages in models with multiple predictors. This approach minimizes the risk of retaining non-significant predictors, as in backward selection, while also avoiding the potential exclusion of relevant predictors due to collinearity issues, which can occur in stepwise regression. Forward selection ensures that only predictors that significantly improve the model are included, reducing overfitting and enhancing model interpretability, which is particularly important in developmental studies examining multiple sociodemographic influences on life satisfaction [26, 27].

Finally, to estimate life satisfaction levels in the participants, percentiles of the global score on a scale of 1 to 100 with 5-point ranges were calculated. The calculation of life satisfaction percentiles was conducted for descriptive purposes, allowing for a clearer interpretation of satisfaction levels within the sample. These percentiles were not used in inferential analyses or predictive models but served to classify participants'life satisfaction scores into interpretable categories. The data were analyzed using SPSS 27.0 and JASP 0.19.0.

## Results

### Descriptive results

According to the results of the Kolmogorov–Smirnov test, none of the SWLS items met the univariate normal distribution assumption (*p's* < 0.001, see Table 1). Additionally, the scale items do not show a multivariate normal distribution (Mardia Skewness = − 1.550—0.662, *p* < 0.0001; Mardia Kurtosis = 2.69—0.148, *p* < 0.0001).

**Table 1:** Descriptive, statistic of normality and proportion for each level of response by item

	Descriptive information							Statistic of Normality		Proportion for each level of response				
	Mean	Std.Dev	Median	Min	Max	Skew	Kurtosis	Lilliefors (KS)	*p* value	1	2	3	4	5
Item_1	3.82	1.01	4.00	1	5	-.806	.297	.256	< 0.001	0.34	0.71	2.09	4.14	2.72
Item_2	4.05	.896	4.00	1	5	− 1.07	1.24	.284	< 0.001	0.16	0.52	1.26	4.78	3.29
Item_3	4.39	.823	5.00	1	5	− 1.55	2.69	.327	< 0.001	0.11	0.21	0.89	3.22	5.57
Item_4	3.70	1.33	4.00	1	5	-.751	-.622	.226	< 0.001	1.03	1.00	1.59	2.67	3.70
Item_5	4.12	.866	4.00	1	5	-.92	.781	.245	< 0.001	0.10	0.37	1.55	4.25	3.74
Total Score	20.08	3.46	20.00	8	25	-.66	.148	.109	< 0.001	–	–	–	–	–

*KS Kolmogorov–Smirnov*

### Confirmatory factor analysis

**Fig. 1:**
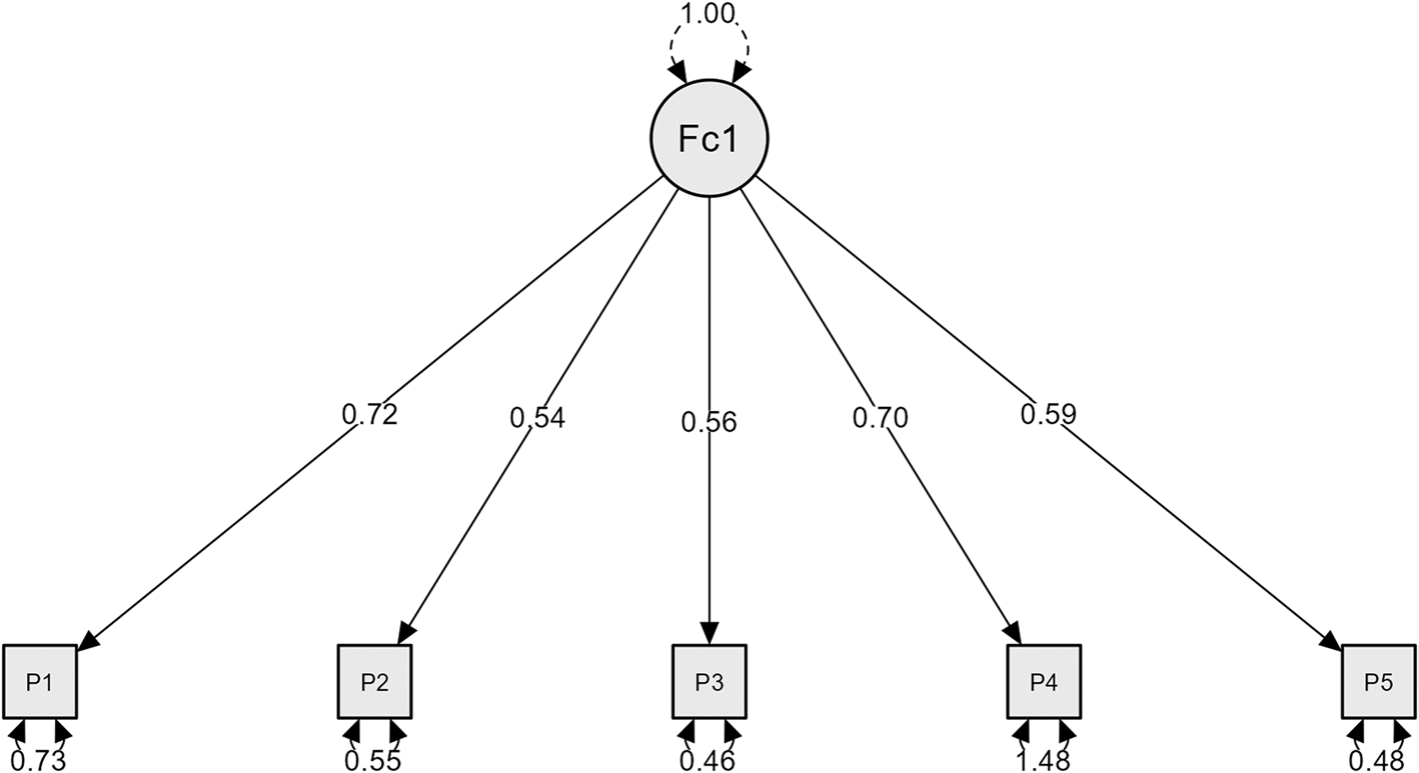
Model 1 Confirmatory Factor Analysis Diagram, one factor of the SWLS-C

The fit of the unidimensional Model 1 of the SWLS corresponding to the five items of the scale was explored using CFA. The Root Mean Square Error of Approximation (RMSEA) of 0.058 suggests an acceptable fit. RMSEA values below 0.05 indicate a good fit, and values between 0.05 and 0.08 suggest a reasonable fit. The 90% CI of RMSEA [0.038–0.081] suggests a reasonable fit, and the p-value for RMSEA (0.234) indicates no significant evidence of poor fit. The Standardized Root Mean Square Residual (SRMR) of 0.019 suggests a very good fit, the Goodness-of-Fit Index (GFI) of 0.999 indicates an excellent fit as values close to 1 are evidence of good fit, the McDonald’s Fit Index (IMF) of 0.992 also suggests an excellent fit as values close to 1 are desirable, and the Expected Cross-Validation Index (ECVI) of 0.045 suggests better predictive fit. In summary, the provided metrics indicate that the instrument has a good fit and is suitable for use based on the presented fit and goodness measures (Table 2 and Fig. 1).

**Table 2:** SWLS-C confirmatory factor analysis index

Index	Model 1	Model 2
Χ^2^	26.15	48.35
Gl	5	13
Comparative Fit Index (CFI)	0.982	0.971
Tucker-Lewis Index (TLI)	0.964	0.956
Root Mean Square Error of Approximation (RMSEA)	0.058	0.047
RMSEA 90% IC Lower Limit	0.038	0.033
RMSEA 90% IC Upper Limit	0.081	0.061
RMSEA p-Value	0.234	0.620
Standardized Root Mean Square Residual (RECMS, SRMR)	0.019	0.023
Goodness of Fit Index (GFI)	0.999	0.999
McDonald's Fit Index (IMF)	0.992	0.986
Expected Cross-Validation Index (ECVI)	0.045	0.066

*Note: χ2* = Normal Theory Weighted Least Squares Chi-Square; gl = degrees of freedom

Model 2 assumes the SWLS-C factor as unidimensional incorporating the influence of age and sex on its fit and goodness indices. In this model, the Chi-square (*X*^2^ = 48.35) indicates a better fit. The Comparative Fit Index (CFI = 0.971), Tucker-Lewis Index (TLI = 0.956), the Root Mean Square Error of Approximation (RMSEA = 0.047), the confidence intervals [0.033–0.061], the Standardized Root Mean Square Residual (SRMR = 0.029), the Goodness-of-Fit Index (GFI = 0.999), McDonald’s Fit Index (IMF = 0.906), and the Expected Cross-Validation Index (ECVI = 0.066) all indicate a good fit.

**Fig. 2:**
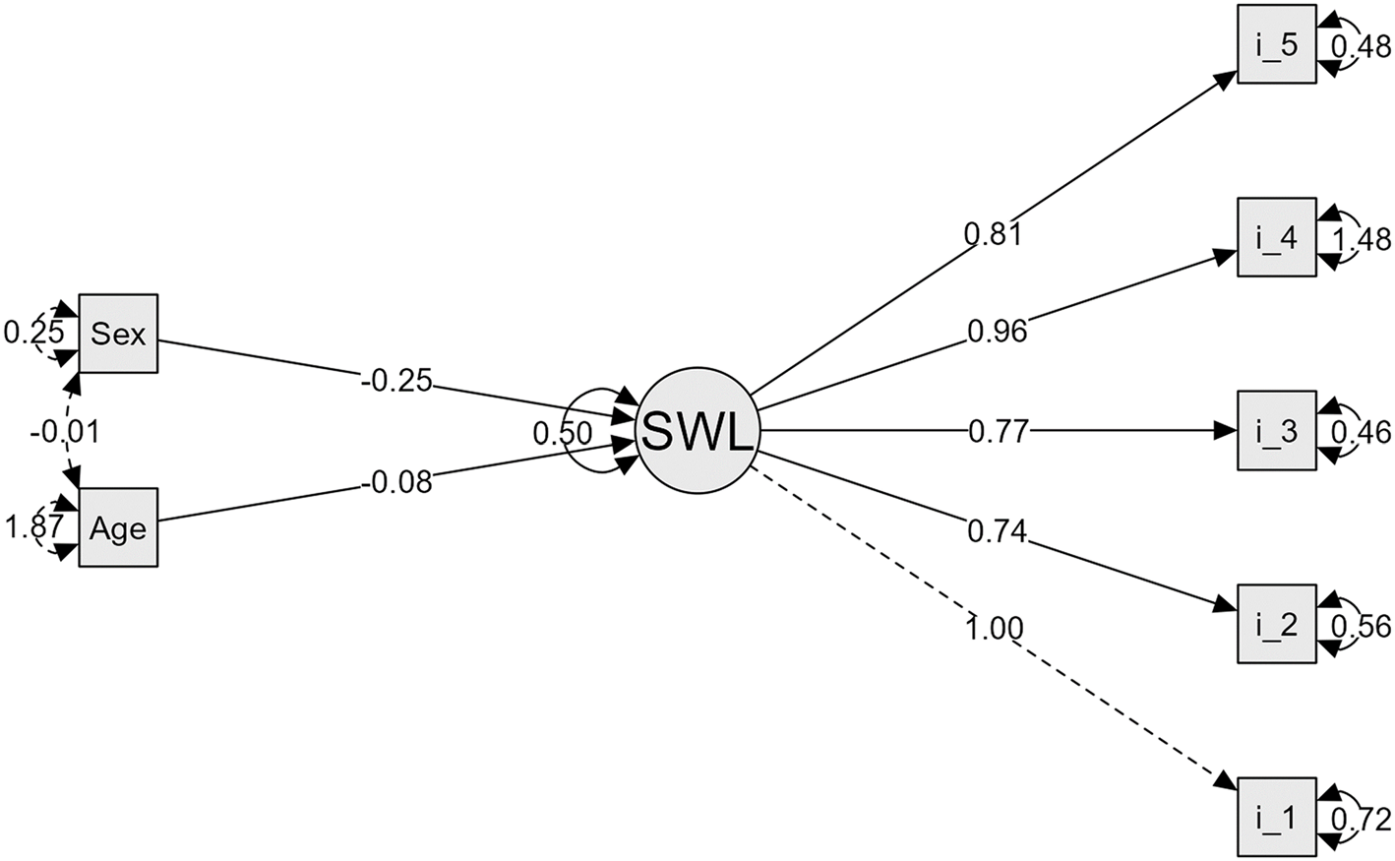
Confirmatory Factor Analysis Diagram, including age and sex of the SWLS-C

The Fig. 2 suggests that the items of the SWLS-C scale are adequately associated with the life satisfaction factor, with additional influences of age and sex that should be considered in the interpretation of the results.

Comparing Models 1 and 2, Model 1 has a lower Chi-square value, indicating a better fit. Both models present good fit indices (CFI, TLI, RMSEA), although Model 1 shows better values overall. The RMSEA and its confidence intervals are good for both models, but Model 2 has a slightly better RMSEA. The GFI values are almost identical and very high for both models, indicating excellent fit. The IMF is better for Model 1, and the ECVI suggests that Model 1 might be preferable in terms of cross-validation. In summary, although both models present a good fit, Model 1 seems to have a slightly better fit based on the Chi-square, CFI, TLI, IMF, and ECVI values.

### Reliability analysis

The reliability analysis of the instrument was calculated using Cronbach's Alpha (α) and McDonald's ω with the overall score of the scale, which included five items. As a unidimensional instrument, the index *α* = 0.721 [95% CI 0.696–0.744] and the index ω = 0.723 [95% CI 0.698–0.747] are considered acceptable.

### Convergent construct validity

The SWLS showed correlations in the expected directions and magnitudes with the other evaluated variables. Moderate correlations were identified with the SLSS scale (0.433**) and perceived social support (0.504**). Additionally, the items of the SWLS scale correlated with the overall score of the SLSS and the Perceived Social Support Scale, as well as their subscales (See Table 3).

**Table 3:** Convergent construct validity of the SWLS-C

	**Family support**	**Friends support**	**Others support**	**Social Support**	**SLSS**
Item 1	.314**	.354**	.281**	.374**	.364**
Item 2	.418**	.346**	.312**	.406**	.303**
Item 3	.416**	.342**	.287**	.390**	.299**
Item 4	.269**	.291**	.241**	.295**	.299**
Item 5	.410**	.393**	.314**	.436**	.331**
Total, Score SWLS-C	.470**	.466**	.387**	.504**	.433**

^**^ All correlation is significant at p ≤ *.005 level*

### Normative data

#### Age, sex, and life satisfaction

The analysis model to establish normative data included age and sex as independent variables. The assumptions considered in the multiple regression analysis were mostly met. There was no multicollinearity 1.055 (VIF values ≤ 3) nor influential cases (maximum Cook's distance = 0.11, indicating the presence of influential cases). The Levene test showed evidence of heteroscedasticity in the model. The standardized residuals of the models were normally distributed (Kolmogorov–Smirnov test). The final regression model showed that scores decrease with age (See Table 4).

**Table 4:** Regression results using F1 as the criterion

Predictor	*b*	*b* 95% CI [LL, UL]	*beta*	*p value*	Fit
(Intercept)	24..90**	[23.16, 26.64]		.000	*R*^*2*^ =.164**
Age	− 0.394*	[− 0.52, − 0.26]	− 0.16	.000	95% CI
Sex	− 0.01**	[− 0.39, 0.36]	− 0.00	.935	

*Note.* A significant *b-weight* indicates the beta-weight and semi-partial correlation are also significant. *b* represents unstandardized regression weights. *beta* indicates the standardized regression weights. *sr*^*2*^ represents the semi-partial correlation squared. *r* represents the zero-order correlation. *LL* and *UL* indicate the lower and upper limits of a confidence interval, respectively. * indicates *p* <.05. ** indicates *p* <.01

Percentile data of the sample according to age and the location of the participants based on these data are presented; the 10-year-old children were mostly located in low and middle-low percentiles (See Tables 5 and 6).

**Table 5:** SWLS-C percentiles according to age

Percentile	10 years	11 years	12 years	13 years	14 years
5	15,00	15,00	14,00	14,00	12,00
10	17,00	16,00	15,00	15,00	13,00
15	17,00	18,00	16,00	16,00	15,00
20	18,00	18,00	18,00	17,00	16,00
25	19,00	19,00	19,00	17,00	17,00
30	19,00	20,00	19,00	18,00	18,00
35	20,00	20,00	19,00	18,00	18,00
40	20,00	20,00	20,00	19,00	19,00
45	20,00	21,00	20,00	19,00	20,00
50	21,00	21,00	21,00	20,00	20,00
55	22,00	22,00	21,00	20,00	20,00
60	22,00	22,00	21,00	20,00	21,00
65	23,00	22,00	22,00	21,00	21,00
70	23,00	23,00	22,00	21,00	22,00
75	23,00	23,00	22,00	22,00	23,00
80	24,00	24,00	23,00	22,00	23,00
85	25,00	24,00	23,00	23,00	24,00
90	25,00	25,00	24,00	23,10	25,00
95	25,00	25,00	25,00	25,00	25,00
100	25,00	25,00	25,00	25,00	25,00
Mean	20,86	20,78	20,14	19,42	19,51
Std Dev	3,037	3,167	3,188	3,259	4,013
Mín	12	10	10	8	8
Máx	25	25	25	25	25

**Table 6:** Distribution of participants according to percentiles SWLS-C

Age	Percentile	F	%
10	Percentile 1–25	74	34,9
	Percentile 25–50	51	24,1
	Percentile 50–75	39	18,4
	Percentile 75–100	48	22,6
	Total	212	100,0
11	Percentile 1–25	60	25,9
	Percentile 25–50	68	29,3
	Percentile 50–75	52	22,4
	Percentile 75–100	52	22,4
	Total	232	100,0
12	Percentile 1–25	66	27,7
	Percentile 25–50	68	28,6
	Percentile 50–75	54	22,7
	Percentile 75–100	50	21,0
	Total	238	100,0
13	Percentile 1–25	79	34,6
	Percentile 25–50	61	26,8
	Percentile 50–75	38	16,7
	Percentile 75–100	50	21,9
	Total	228	100,0
14	Percentile 1–25	97	29,2
	Percentile 25–50	98	29,5
	Percentile 50–75	75	22,6
	Percentile 75–100	62	18,7
	Total	332	100,0

## Discussion

The present study examined the psychometric properties of the Satisfaction with Life Scale for Children (SWLS-C) in a sample of 1,242 Colombian children. The findings indicate that the SWLS-C is a valid and reliable instrument for assessing life satisfaction in this population, demonstrating a good fit to the unidimensional model and acceptable internal consistency. These results align with previous research on the SWLS-C and the subjective well-being theory [14–16, 20, 28].

First, confirmatory factor analysis (CFA) showed that the unidimensional model of the SWLS-C exhibited an adequate fit, with an RMSEA of 0.058 (90% CI [0.038, 0.081]), an SRMR of 0.019, and a GFI of 0.999. These findings are comparable to those reported in previous studies [10], which found high internal consistency and good fit indices in a sample of Canadian children. Similarly, research conducted in other cultural contexts, such as Chile [1] and Indonesian [20], has also demonstrated good model fit and high internal consistency for the SWLS-C, with CFA indices including RMSEA values below 0.05 and CFIs above 0.95.

The internal consistency of the SWLS-C in the present study, with a Cronbach's alpha of 0.721 and McDonald's omega of 0.723, while slightly lower than in other studies, remains acceptable and falls within the range reported in similar research, where alpha values between 0.84 and 0.93 have been documented [11, 13].

In terms of convergent validity, the moderate correlations found between the SWLS-C and other measures of well-being, such as the SLSS (r = 0.433) and the Zimet Perceived Social Support Scale (r = 0.504), support the construct validity. These results align with previous studies that have found significant correlations between life satisfaction and perceived social support [18, 29–31] Additional have consistently demonstrated that life satisfaction correlates with various indicators of subjective well-being, further supporting the current results [3, 32].

The results of this study provide evidence for the convergent validity of the SWLS-C, as indicated by its significant positive correlation with perceived social support (r = 0.504, p < 0.01). This moderate correlation aligns with theoretical expectations, as social support has been consistently identified as a key factor in subjective well-being, particularly in childhood and adolescence. The relationship suggests that children who perceive stronger support from their family, friends, and significant others also tend to report higher life satisfaction, reinforcing the conceptual validity of the SWLS-C as a measure of subjective well-being.

Regarding convergent validity, moderate correlations were identified between the SWLS-C and other well-being measures, such as the SLSS scale (r = 0.433) and the Zimet Perceived Social Support Scale (r = 0.504), supporting its construct validity. These findings align with theoretical expectations, as social support has consistently been recognized as a key factor in subjective well-being, particularly during childhood and adolescence. The observed relationship suggests that children who perceive greater support from their family, friends, and significant others also tend to report higher life satisfaction, further reinforcing the conceptual validity of the SWLS-C as a measure of subjective well-being [18, 29–31]

These findings are consistent with previous research indicating that social support plays a crucial role in psychological adjustment and overall life satisfaction. Since childhood and adolescence are developmental periods characterized by a high reliance on social relationships, the perception of a strong support network may act as a buffer against stress, fostering a more positive evaluation of life.

The moderate strength of this correlation suggests that, while social support is an important determinant of life satisfaction, other psychological and contextual factors may also play a significant role in shaping children's well-being.

Future research should further explore the interaction between social support and life satisfaction, incorporating longitudinal analyses to assess whether perceived social support predicts changes in life satisfaction over time. Additionally, examining the influence of different types of social support (e.g., family support vs. peer support) on subjective well-being could provide deeper insights into how social networks contribute to well-being during childhood[12, 18].

The internal consistency coefficient obtained in this study was slightly lower than that reported in previous research [10, 13], which may be attributed to cultural and contextual differences. Specifically, perceptions of life satisfaction may vary across populations due to socioeconomic factors, family structures, and cultural values, potentially explaining the differences in SWLS-C scores between Colombian children and those from countries such as Canada, South Korea, and Norway[14, 17]

This study confirms the psychometric validity of the SWLS-C in Colombian children, with fit indices that align with previous studies conducted in Spain and Portugal [12]. While the fit indices in the Spanish and Portuguese samples were nearly perfect (CFI = 1.00 in Spain, CFI = 0.998 in Portugal), our study obtained slightly lower but still acceptable values (CFI = 0.982, RMSEA = 0.058). These differences may be attributed to cultural variations, linguistic nuances, or sample characteristics.

Additionally, the study conducted in Spain and Portugal reported issues with scalar invariance, suggesting that adolescents from different cultural backgrounds may interpret life satisfaction items differently [12]. Although factorial invariance was not assessed in the present study, similar challenges may arise in the Colombian context, particularly among different socioeconomic groups.

This study provides evidence of the psychometric validity of the SWLS-C in Colombian children, with fit indices comparable to previous studies conducted in Spain and Portugal [12]. While the Spanish and Portuguese samples reported near-perfect fit indices (CFI = 1.00 in Spain, CFI = 0.998 in Portugal), our study obtained slightly lower but still acceptable values (CFI = 0.982, RMSEA = 0.058). These differences may be attributed to cultural variations, linguistic nuances, or sample characteristics.

Additionally, the study conducted in Spain and Portugal reported issues with scalar invariance, suggesting that adolescents from different cultural backgrounds may interpret life satisfaction items differently [12]. Although factorial invariance was not assessed in the present study, similar challenges may arise in the Colombian context, particularly among different socioeconomic groups. Given that Spanish-speaking countries share linguistic similarities and certain idiosyncratic traits, factors such as quality of life and unmet basic needs—which are more prevalent in countries like Colombia—could influence well-being perceptions. In societies with higher economic stability, life satisfaction assessments may reflect personal achievements and self-fulfillment, whereas in developing countries, well-being perceptions may be more closely linked to financial security, access to basic services, and social protection.

This study has several limitations that should be considered when interpreting the results. Firstly, the sample was limited to Colombian children, which may limit the generalizability of the findings to other populations. Future research should include more diverse and representative samples from different cultural and socioeconomic contexts to validate the SWLS-C more broadly. China [33], Turkey [18] and Canada [14] are exemplifies the importance of conducting cross-cultural studies to ensure the validity of the scale in various contexts.

Therefore, future studies should examine measurement invariance to determine whether the SWLS-C functions equivalently across different demographic subgroups in Colombia. Analyzing factorial invariance by gender would help establish whether boys and girls interpret life satisfaction items consistently, or if cultural expectations and socialization processes lead to systematic differences in responses. Previous research suggests that gender norms may influence self-reported well-being, with girls often reporting lower life satisfaction due to higher emotional awareness or greater sensitivity to social comparisons. If scalar invariance is not met, it could indicate that observed gender differences reflect measurement biases rather than actual disparities in life satisfaction[12].

Similarly, evaluating age-related measurement invariance would provide insight into whether life satisfaction constructs remain stable across developmental stages. Since cognitive maturation and social experiences evolve between ages 10 and 14, differences in how younger children and older adolescents interpret abstract concepts such as happiness and life satisfaction may emerge. If full measurement invariance is not achieved across age groups, this would suggest that developmental factors influence how children conceptualize well-being, which has implications for longitudinal studies tracking life satisfaction changes over time.

Future research should prioritize testing for configural, metric, and scalar invariance to ensure that comparisons between gender and age groups are meaningful. This would contribute to a more precise interpretation of whether differences observed in life satisfaction scores stem from actual variations in well-being or measurement inconsistencies. Additionally, exploring socioeconomic-level invariance could provide further insights into how contextual disparities shape children’s subjective well-being perceptions.

Another important consideration is the influence of sociocultural and economic factors on life satisfaction assessments in Colombian children. Although life satisfaction scores in this sample were slightly lower compared to other countries such as Spain, Portugal, and Chile, this may be attributed to differences in quality of life, access to resources, and socioeconomic security. Previous studies have indicated that in countries with greater economic inequality, well-being perceptions may be more strongly influenced by unmet basic needs, such as access to healthcare, quality education, and safety. In this sense, in countries like Colombia, where socioeconomic disparities are more pronounced, life satisfaction may be evaluated using a different frame of reference than in nations with greater economic stability.

Previous research has shown that in socially vulnerable contexts, expectations regarding quality of life may be conditioned by experiences of material deprivation or limited access to opportunities, influencing how children assess their subjective well-being [34]. Given this context, future studies should further examine how socioeconomic and cultural conditions impact life satisfaction measurement in Latin American children, assessing whether the SWLS-C maintains its structural validity in different vulnerable settings.

The evaluation of factorial invariance among socioeconomic groups within Colombia would help determine whether the scale measures life satisfaction equivalently across children from different economic backgrounds, or if observed differences reflect measurement biases.

Another area for future research could focus on the predictive validity of the SWLS-C, examining how scores on this scale predict important long-term outcomes, such as academic performance, mental health, and general well-being in adolescence and adulthood. For example, longitudinal studies have shown that life satisfaction in childhood can significantly impact psychological well-being in adulthood, highlighting the importance of measuring and promoting subjective well-being from early ages [35].

The results of this study provide strong evidence that the SWLS-C is a valid and reliable instrument for assessing life satisfaction in Colombian children. The findings are consistent with the theory of subjective well-being and previous studies conducted in other cultures, although some minor differences underscore the importance of considering cultural and contextual influences in the evaluation of life satisfaction [36, 37].

Despite these considerations, one of the key strengths of this research is the rigorous evaluation of the psychometric properties of the SWLS-C in a large sample of Colombian children, providing valuable evidence of its validity and reliability in a Latin American context. Unlike previous studies that have examined this scale in economically stable countries, this research offers insight into its applicability in a population characterized by greater socioeconomic disparities.

Furthermore, the inclusion of diverse analyses, such as confirmatory factor analysis (CFA) and convergent validity evaluation, reinforces the methodological robustness of the study and strengthens conclusions about the scale's utility. Another notable aspect is the consideration of sociodemographic variables, such as age and gender, which lays the groundwork for future studies on the factorial invariance of the SWLS-C across different subgroups of the child population.

Finally, beyond its psychometric contributions, this research has practical implications, as its findings could inform policies and intervention strategies aimed at enhancing child well-being in socially and economically vulnerable contexts.

In terms of practical applications, these findings highlight the importance of developing public policies aimed at reducing structural inequalities that impact child well-being. The integration of the SWLS-C in school evaluations and national well-being monitoring programs could be a valuable strategy to identify groups of children at risk of lower life satisfaction and design interventions tailored to their needs.

Additionally, community-based programs that strengthen social support networks and protective relationships in childhood could be key to improving life satisfaction levels in vulnerable populations.

In conclusion, this study confirms the psychometric validity of the SWLS-C in the Colombian child population and emphasizes the importance of considering cultural, economic, and social factors in the evaluation of subjective well-being. While the findings demonstrate that the scale is a valid and reliable tool in this context, further research is needed to understand how structural inequalities and differences in well-being conceptualization may affect its application in diverse cultural and socioeconomic settings.

### Ethics committee

The data for this manuscript were collected and handled in accordance with the local ethics committee of the Universidad del Sinú, on April 30, 2024, with record number 003.

## Supplementary Information

Supplementary Material 1.

## Data Availability

The datasets used and analysed during the current study will be available from the corresponding author on reasonable request.
