# Supplementary material for: Life satisfaction in children: an analysis of the psychometric properties of the SWLS-C scale by Diener
Source: BMC Psychol. 2025 Apr 15;13:383. doi: 10.1186/s40359-025-02690-4 (PMC11998223; doi:10.1186/s40359-025-02690-4)
Supplement: Supplementary file 1 — Supplementary Material 1. [file 40359_2025_2690_MOESM1_ESM.pdf]

EL COMITÉ DE ÉTICA INSTITUCIONAL DE LA UNIVERSIDAD DEL SINÚ
CERTIFICA:

Que el proyecto de investigación titulado "***Eficacia de un modelo de intervención basado en la gratitud, el perdón, el orgullo y la empatía sobre la salud mental en niños entre 8 y 12 años del departamento de Córdoba***" , presentado por: Marly Johana Bahamón (investigadora Principal) , José Julián Javela González y Andrea Ortega Bechara (COINVESTIGADORES) , del grupo de investigación I-FLOR de la Universidad del Sinú, fue evaluado y aprobado por este comité en su sesión del día 30 de abril de 2024, considerando la pertinencia de la investigación, el rigor metodológico, su calidad científica y el cumplimiento de las Normas Científicas, Técnicas y Administrativas para la Investigación en Salud establecidas en la Resolución No. 008430 de 1993.

El Comité de Ética Institucional de la Universidad del Sinú avala que el proyecto cumple con todos los requisitos de calidad exigidos y le otorga su aprobación, como aparece en el acta N° 003 del 30 de abril de 2024

Se expide esta certificación el 03 de mayo de 2024.

ÁLVARO BUSTOS GONZÁLEZ
Presidente del Comité de Ética Institucional Universidad del Sinú – Elías Bechara Zainum
